# Reviewed article: Araujo MC, Passinho RS, Pereira RSF, Mora DJ. Temporal trends in incidence and mortality from pulmonary tuberculosis: time series study, Southern Bahia, Brazil, 2010-2023. Epidemiol Serv Saude. 2025:34;e20240778

**DOI:** 10.1590/S2237-96222025v34e20240778.c

**Published:** 2025-08-04

**Authors:** Jefferson Felipe Calazans

**Affiliations:** Universidade Tiradentes

**Table:** 

Rating	Excellent	Good	Average	Below average	Poor
Interest			✓		
Quality		✓			
Originality			✓		
Overall			✓		

**Table:** 

Questionnaire	Yes	No	Not applicable
Does the manuscript contain new and significant information to justify publication?	✓		
Does the Abstract (Summary) clearly and accurately describe the content of the article?	✓		
Is the problem significant and concisely stated?		✓	
Are the methods described comprehensively?	✓		
Are the interpretations and conclusions justified by the results?	✓		
Is adequate reference made to other work in the field?		✓	

## Manuscript Structure

Length of article is: Adequate

Number of tables is: Too few

Number of figures is: Adequate

Please state any conflict(s) of interest that you have in relation to the review of this paper (state “none” if this is not applicable): None

## Comments

Título:

- Sugiro inseir o tipo de estudo no título

- É preferível, no período: “no período de 2010 a 2023” OU simplesmente “2010-2023”

- O correto seria da incidência e mortalidade POR tuberculose pulmonar

- Acredito que sexo e faixa etária pode ser dispensável do título

- Não seria “na região sul da Bahia”?

Resumo:

- Adequar objetivo conforme alterações no título

- Se trata de um estudo de série temporal, mas o tipo não foi adequadamente descrito. Trata-se de um estudo ecológico de série temporal. Adequar isso no resumo e no método do trabalho

- Não seria estudo da incidência e mortalidade e sim um estudo sobre tuberculose pulmonar no qual analisou os casos e mortes e posteriormente calculou incidência e mortalidade. Adequar.

- Na Costa do Descobrimento e Extremo Sul DA BAHIA

- Corrigir: intervalo de confiança (IC95%) para “intervalo de confiança a 95% (IC95%)”

- “e a mortalidade, 1,1 óbitos por 100 mil habitantes” não seria: “e a mortalidade de 1,1 óbitos por 100 mil habitantes”

- Ajustar as casas decimais em todo o trabalho: Percentuais (VPA e afins) 1 casa decimal, taxas 2 casas decimais, p-valor três casas decimais. Compreendo que os valores da VPA são ínfimos, se o periódico concordar pode manter duas casas decimais.

- O correto não seria: “ocorreram NO sexo feminino”?

- Padronizem a menção do VPA entre os parênteses: (VPA: 0,01; IC95%: 0,01;0,02)

- Essa frase: “Descobrimento apresentou incidência decrescente para o sexo feminino e a faixa etária de 40 a 59 anos, enquanto o Extremo Sul, de 20 a 39 anos.” Ficou bastante confusa, sugiro reescrever. Além disso, é mencionado tendência decrescente, mas a VPA não foi descrita. Todo o resultado escrito no resumo precisa dos valores numéricos, sugiro ‘sacrificar’ outros resultados menos relevantes se for necessário.

- A conclusão deve somente responder ao objetivo proposto.

Introdução:

- No parágrafo que inicia na linha 52 da página 3, é necessário reorganizar as ideias, separando os achados do maior nível geográfico para o menor nível.

- O parágrafo seguinte (da pandemia) aparenta estar solto, não compreendi como esse parágrafo agrega ao estudo, uma vez que não faz parte do seu objetivo analisar influências do período pandêmico. Entendo que esse período houve problemas na notificação, mas isso seria mais bem exposto nas limitações.

- Na linha 10 da página 4 é descrito sobre populações vulneráveis. Estudar sexo e faixa etária não indica o estudo de populações vulneráveis então a afirmação não caiu bem.

- A justificativa do estudo é rasa. Por que seu estudo é relevante? (somente verificar quem é mais afetado não é suficiente), como o trabalho se diferencia dos demais publicados? Como um estudo de série temporal pode ajudar? Por que estudar a Bahia? Por que estudar o SUL da Bahia? Essas são perguntas que os autores podem fazer para melhorar a justificativa do trabalho.

Métodos:

- Adequar o delineamento ao que foi dito no resumo.

- Delineamento não deve constar o tipo de dado, local ou variáveis. Somente características do estudo. O local e período é no Contexto, as variáveis são em Variáveis.

- Participantes tem informação do local repetida, sugiro manter somente no local apropriado.

- Na seção participantes, é necessário descrever também todo e qualquer procedimento relacionado à seleção dos participantes. A exemplo, é preciso que os autores descrevam o que foram considerados como casos novos. Tudo que foi realizado para chegar ao N do estudo deve ser descrito.

- Variáveis: deve ser descrito a variável e seu agrupamento original a exemplo: sexo (masculino, feminino, ignorado), situação de encerramento do caso (cura, recidiva...)

- Se foi utilizado idade detalhada para transformar em faixa etária, essa menção deve ser apontada na seção “Detalhamento das variáveis” ou “Variáveis quantitativas” (conforme RECORD).

- Os dados foram obtidos do SINAN disponibilizados pela Secretaria... isso passa a impressão de que foram utilizados dados primários, coletados da secretaria do estado, cuja natureza é identificável, logo, passível de aprovação do comitê de ética. Mas, é dito ao final que se trata de um banco de dados público. Sugiro então, apontar que os dados foram extraídos do SINAN, disponibilizados pelo DATASUS, acessados via Tabnet (OU Tabwin). Adicionem também o mês e ano do levantamento, bem como, como foi realizado o levantamento de forma breve (ou usando um fluxograma).

- Cálculo de incidência e mortalidade é descrito na seção de variáveis, pois essas são variáveis.

- Na fonte de dados deve ser mencionado não só o SINAN mas também o IBGE, bem como, como foi o acesso e o período de acesso.

- Transformação logarítmica deve ser descrito na análise dos dados, pois ela é necessária somente para a regressão de Prais-Winsten.

- Descrição da dispensa do comitê de ética deve ser colocado como uma seção separada, após a seção de métodos estatísticos.

- Controle de viés é sim aplicável. A técnica de Prais-Winsten é um motivo de controle de viés, por corrigir a autocorrelação na série temporal.

- A seção “tamanho do estudo” é aplicável. Geralmente, a tendência é escrever o tamanho do estudo no início dos resultados, portanto, podem remover a seção.

- Um ponto MUITO IMPORTANTE: Os dados dos óbitos foram retirados da variável encerramento do caso, mas para o delineamento proposto não seria o ideal. Os dados do Sistema de Mortalidade são melhores e contam com muito menos subnotificações. No delineamento proposto é perfeitamente possível analisar os dados do SIM, então sugiro uma reformulação nesse quesito, com a realização de novas taxas, tendências e tudo que for relacionado à mortalidade.

Resultados:

- Não repetir valores que estão presentes na tabela em texto. Revisar todo o resultado para adequar isso.

- Lembrar de padronizar as casas decimais.

- Sintetizar o texto dos resultados, há muito texto, quase tudo que foi observado foi descrito junto com seus valores numéricos. A seção está densa e atrapalha a leitura. Geralmente, é comum escrever o que é mais importante de achado e que geralmente vai ser discutido no futuro. Escrevam o resultado com base na avaliação de vocês e que auxiliará o leitor a entender o que foi encontrado de mais importante. Prezem por qualidade e não quantidade.

- Alguns parágrafos não fazem menção à sua respectiva tabela.

- Sugiro que a ordem dos resultados sejam: dados gerais da população estudada, dados das taxas, resultados da tendência. Falando primeiro da incidência depois da mortalidade. Ficará melhor para leitura comparado a forma original onde há dois grandes blocos de resultado um para incidência e um para mortalidade.

Discussão:

- Primeiro parágrafo: deve refletir os principais achados do estudo, fazendo uma sintetização, de preferência sem repetir números, podendo interpretar os achados. Sugiro reformular, o que foi feito não reflete os resultados encontrados. Cuidado com a afirmação “seria a mais vulnerável” pode ser muito subjetiva.

- Segundo parágrafo: Qual o período dos dados que foram comparados? Assegurem ao máximo comparações 1:1. Notei que as citações são boletins epidemiológicos que geralmente analisam meio período ou um ano completo. Os dados apresentados no estudo são médias de mais de 10 anos. Seria interessante estudos semelhantes que também utilizaram média. Além disso, após a comparação do resultado os autores escrevem sugestões para melhoria, sem discutir o porquê do achado encontrado, fato imprescindível em uma discussão. Toda a discussão do trabalho deve ser baseada nos blocos: 1) chamativa do resultado a ser discutido, interpretando e preferencialmente sem repetir número; 2) comparação com resultados da literatura; 3) justificativa do resultado encontrado ou da comparação realizada; 4) sugestões de melhoria, hipóteses autorais etc. Isso se repete para cada resultado a ser discutido. Adequar a todos os parágrafos da discussão.

- Terceiro parágrafo: iniciado por um resultado da literatura, a ordem está errada iniciar pelo resultado encontrado na pesquisa.

- Limitações do estudo: falácia ecológica é um viés em estudos ecológicos, mas quando cometido. Acredito que tal limitação não seja interessante uma vez que os resultados estudados não foram extrapolados a nível individual. Existem outras limitações do estudo que podem ser exploradas, além da subnotificação, como a pandemia da COVID-19, a baixa população estudada que pode influenciar nas estimativas de tendência, a pouca representatividade do estudo por estudar somente uma região em um estado do Brasil, não ter considerado outras variáveis demográficas como raça/cor, escolaridade ou variáveis clínicas que podem melhor explicar alguns fenômenos etc.

- Após as limitações precisam ser descritas as potencialidades do estudo, o que ele tem de positivo. Falem bem do estudo de vocês. Cuidado somente com exageros como: “irá ajudar a reduzir os casos e mortes por tuberculose”

- O último parágrafo da discussão deve ser a conclusão do estudo, que responde puramente aos objetivos propostos, sem repetir números, sem interpretações.

Considerações gerais

- Na tabela 1 o correto é o título Faixa etária e não Idade

- Adequar as casas decimais da tabela 1

- Inserir que as taxas são por 100 mil

- É imprescindível, uma vez que é possível, inserir o p-valor exato na tabela 2 usando 3 casas decimais

- Há espaço para inserir uma tabela com as taxas por ano e localidade estudada, elas são mencionadas em textos mas não aparecem em tabela

- Toda média deve ser sucedida do seu desvio padrão, adequar isso em todo o texto e tabela, se for o caso. 

